# Pulmonary artery augmentation and aortic valve repair using novel tissue-engineered grafts

**DOI:** 10.1016/j.xjtc.2021.09.058

**Published:** 2022-01-21

**Authors:** Hisayuki Hongu, Masaaki Yamagishi, Keiichi Kanda, Yoshinobu Maeda, Tomoya Inoue, Hiroki Nakatsuji, Hitoshi Yaku

**Affiliations:** a Department of Pediatric Cardiovascular Surgery, Children's Medical Center, Kyoto Prefectural University of Medicine, Kyoto, Japan; b Division of Cardiovascular Surgery, Kyoto Prefectural University of Medicine, Kyoto, Japan

**Keywords:** tissue-engineered vascular grafts, autologous pericardium, pulmonary artery, aortic valve plasty, pediatric cardiovascular surgery, AVP, aortic valve plasty, PA, pulmonary artery, RVOT, right ventricular outflow tract, TEVG, tissue-engineered vascular graft

## Abstract

**Objectives:**

The objectives of this study were to evaluate the results when tissue-engineered vascular grafts (TEVGs) are used as alternatives to autologous pericardium for surgically augmenting the pulmonary artery (PA) or aortic valve.

**Methods:**

TEVG molds were embedded into subcutaneous spaces for more than 4 weeks preoperatively. Since 2014, 6 patients have undergone PA reconstruction, whereas 1 has undergone aortic valve plasty (AVP) with TEVGs. The time from mold implantation to the operation was 8.9 (range, 6.0-26.4) months. The age and body weight at the time of operation were 2.7 (range, 1.8-9.2) and 11.6 (range, 7.9-24.4) kg, respectively. Concomitant procedures comprised the Rastelli, palliative Rastelli, and Fontan operations in 2, 2, and 1 patient, respectively.

**Results:**

The median follow-up period was 14.4 (range, 3-39.6) months. There were no early or late mortalities. Moreover, there were no TEVG-related complications, including aneurysmal changes, degeneration, and infection. In 5 patients who underwent PA augmentation, the postoperative PA configuration was satisfactorily dilated. The reconstructed aortic valve function was good in the patient who underwent AVP. Decreased leaflet flexibility due to leaflet thickening was not observed. One patient had postoperative PA re-stenosis; therefore, re-PA augmentation with TEVGs was performed. On histological examination, TEVGs consisted of collagen fibers and few fibroblasts, and elastic fiber formation and/or smooth muscle cells were not observed.

**Conclusions:**

The midterm results of PA reconstruction and AVP with TEVGs were satisfactory. TEVGs might be a useful alternative to autologous pericardium in pediatric cardiovascular surgeries that often require multistage operations.

## Graphical abstract

Our novel technique of the tissue-engineered vascular graft using for pulmonary artery augmentation and aortic valvuloplasty.

**Figure:**
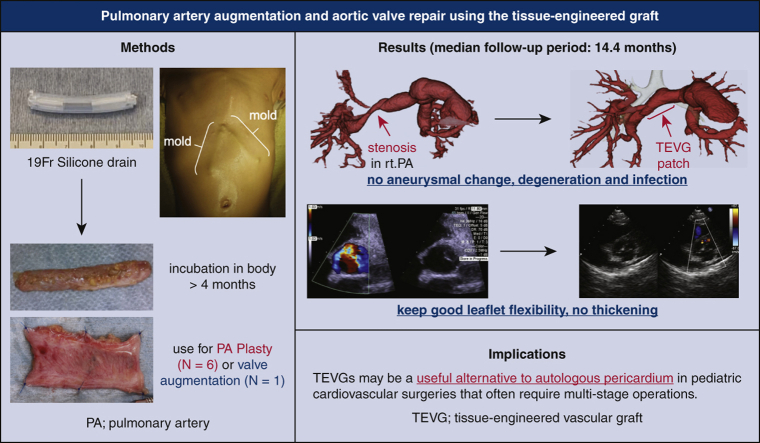

**Figure:**
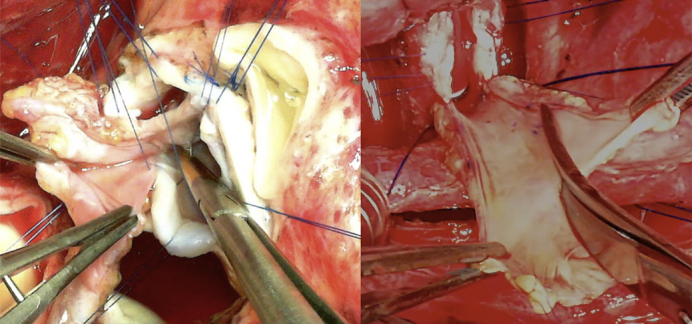
Clinically used tissue-engineered vascular graft patch.

**Central Message**

Midterm outcomes of pulmonary artery reconstruction and aortic valve plasty with tissue-engineered vascular grafts were satisfactory. Thrombosis, aneurysmal change, degeneration, and infection were absent.

**Perspective**

Our tissue-engineered vascular grafts might be useful alternatives to autologous pericardium in pediatric cardiovascular surgeries, such as pulmonary artery augmentation, valve leaflet augmentation, and surgeries requiring multiple stages.

Autologous pericardium is a material conventionally used in pediatric cardiovascular surgeries, especially for pulmonary artery (PA) surgical dilation and/or valve leaflet augmentation. As opposed to other materials such as artificial grafts, homografts, and xenografts, autologous pericardium is resistant to infection and calcification, does not provoke autoimmune responses, and has growth potential. However, the utility of autologous pericardium is limited because of various reasons, including adhesion effects after multiple thoracotomy or mediastinum inflammation and exhaustion in previous surgeries.^1^ Since 2014, we have been developing tissue-engineered vascular grafts (TEVGs) as alternatives to autologous pericardium for use in congenital heart surgeries, and have reported good results for the same.^2^, ^3^, ^4^ In this study we aimed to investigate the midterm outcomes of PA reconstruction and aortic valve plasty (AVP) with TEVGs.

## Methods

This study was approved by the institutional review board of the Kyoto Prefectural University of Medicine (approval number: ERB-C-162; approval date: May 12, 2014). Written informed consent was obtained from the patients' parents or guardians for the publication of their data.

### Methods

**Figure 1:**
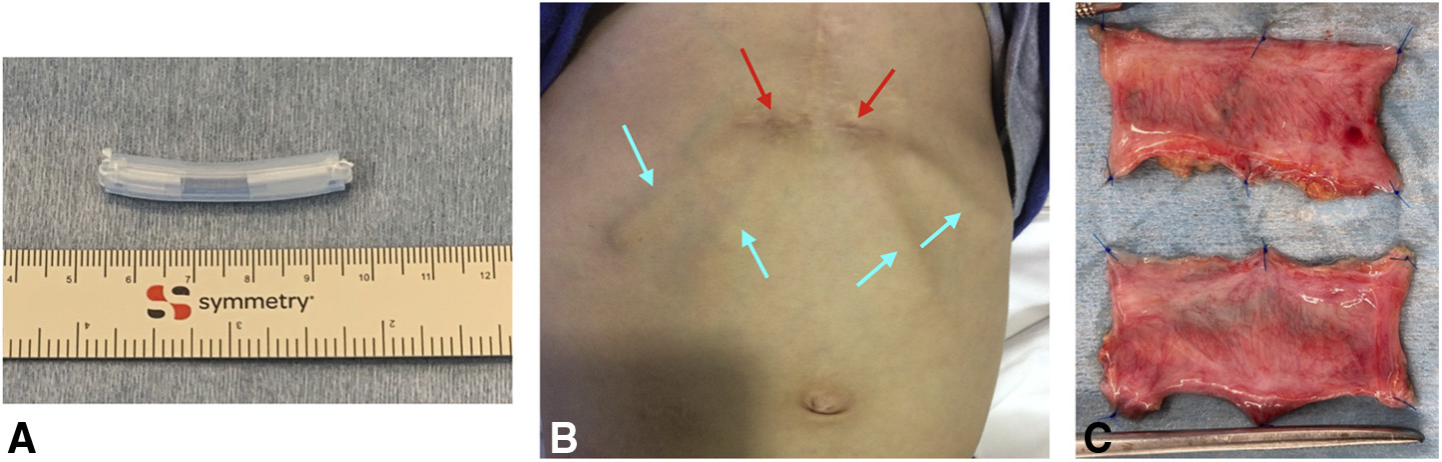
Images of the tissue-engineered vascular graft. A, A tissue-engineered vascular graft mold made with a 19-French silicone drain tube. B, Postoperative image of the local skin incision (*red arrows*) and 4 molds implanted in the subcutaneous spaces (*blue arrows*). C, Opened tissue-engineered vascular grafts.

TEVG molds were made from 19-French silicone drain tubes in a manner that ensured that there were no lumina in the molds (Figure 1, *A*). The molds were subsequently embedded in the upper abdominal subcutaneous space (Figure 1, *B*) either independently before the surgery or in a concomitant procedure at the time of previous surgeries. In each patient, we were able to insert the mold through a small skin incision that was a few centimeters wide. After more than 4 months after the implantation, the TEVGs were harvested intraoperatively (Video 1) and treated with 70% ethanol for 10 to 15 minutes while observing the TEVG strength; during harvesting, a skin incision was made directly above the mold to avoid damage to the TEVGs. The treated TEVGs were then used as patch materials by cutting them open (Figure 1, *C*).

**Video 1:**
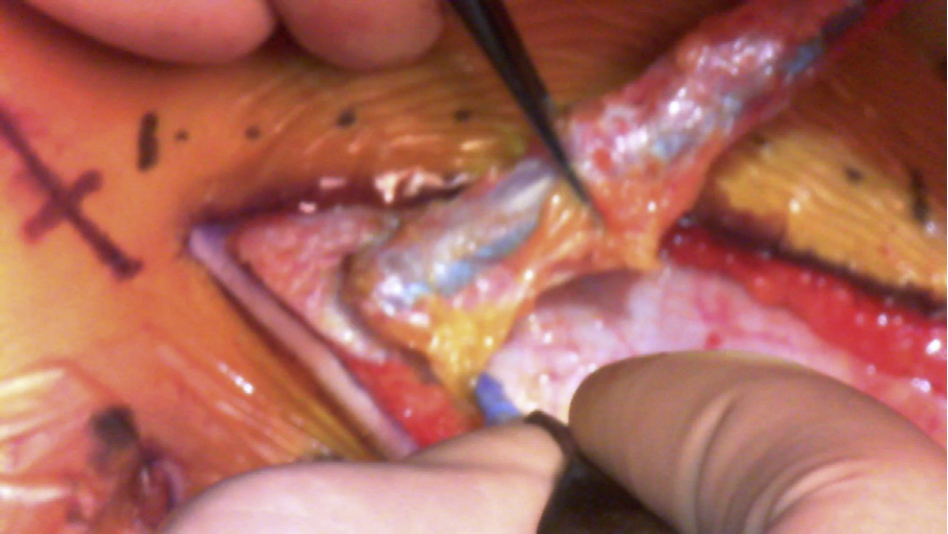
Harvest of tissue-engineered vascular graft mold. Video available at: https://www.jtcvs.org/article/S2666-2507(22)00016-5/fulltext.

### Data Collection and Analysis

Clinical data were obtained from a retrospective review of medical records and operative and echocardiographic reports. Frequencies are presented as absolute numbers and percentages. Continuous data are presented as medians with ranges or as means with SDs.

### Patients

**Figure 2:**
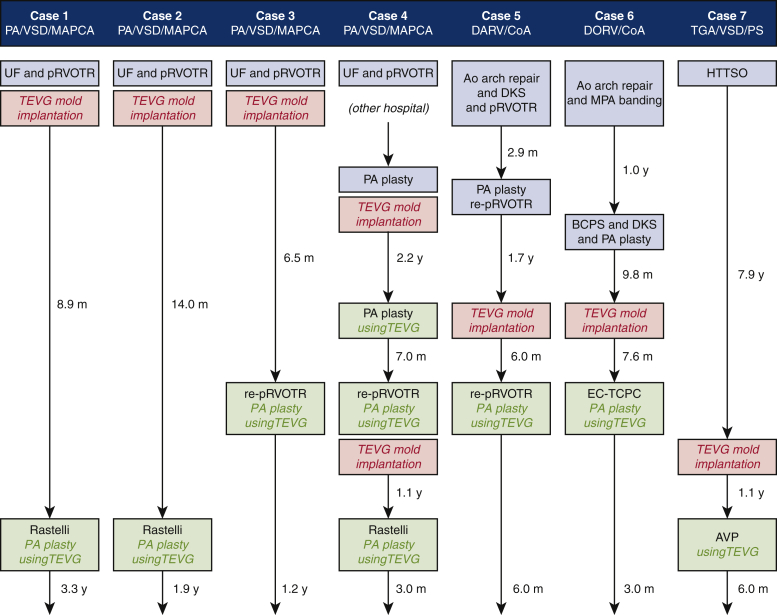
The profile of all 7 patients. *PA*, Pulmonary atresia; *VSD*, ventricular septal defect; *MAPCA*, major aortopulmonary collateral arteries; *DORV/CoA*, double-outlet right ventricle/coarctation of the aorta; *TGA*, transposition of the great arteries; *PS*, pulmonary stenosis; *UF*, unifocalization; *pRVOTR*, palliative right ventricular outflow tract reconstruction; *DKS*, Damus–Kaye–Stansel anastomosis; *MPA*, main pulmonary artery; *HTTSO*, half-turned truncal switch operation; *TEVG*, tissue-engineered vascular graft; *PA*, pulmonary artery; *BCPS*, bidirectional cavopulmonary connection; *EC-TCPC*, extracardiac total cavopulmonary connection; *AVP*, aortic valve plasty.

**Figure 3:**
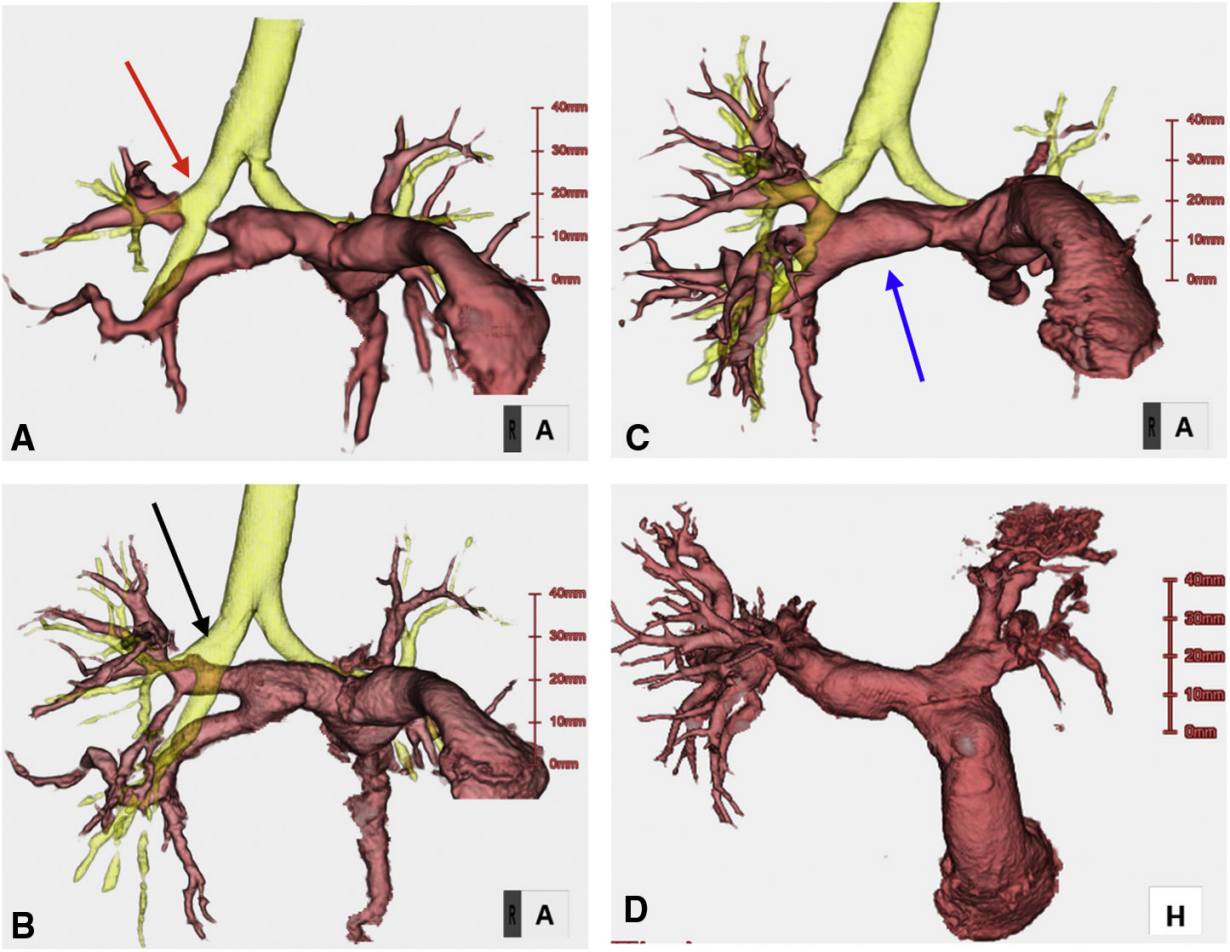
Pre- and postoperative 3-dimensional cardiac computed tomography scan of patient 4. A, Image taken after the first pulmonary artery augmentation with autologous pericardium. The distal right pulmonary artery is compressed by the right bronchus (*red arrow*). B, Image taken after pulmonary artery augmentation with tissue-engineered vascular graft patch for the first time. This patch was implanted at the stenotic region of the pulmonary artery (*black arrow*). C and D, Images taken after the final Rastelli operation. In the Rastelli operation, the graft was used again for the pulmonary artery (*blue arrow*).

Since July 2014, 6 patients and 1 patient have undergone PA reconstruction (Video 2) and AVP (Video 3) with TEVGs at our center, respectively (Figure 2; Tables E1, and E2). The diagnoses comprised pulmonary atresia, ventricular septal defect, and major aortopulmonary collateral arteries in 4 patients (group A); double-outlet right ventricle and coarctation of the aorta in 2 patients (group B); and transposition of the great arteries, ventricular septal defect, and left ventricular outflow tract obstruction in 1 patient (the AVP patient, group C). The median time from mold implantation to operation was 8.9 (range, 6.0-26.4) months. The median age and body weight of the patients at the time of the operation were 2.7 (range, 1.8-9.2) years and 11.6 (range, 7.9-24.4) kg, respectively. In group A, TEVG was embedded in 3 patients during the first palliative surgery (unifocalization with palliative right ventricular outflow tract [RVOT] reconstruction). Of these patients, 2 received TEVG for PA augmentation during the next Rastelli operation, whereas 1 did not meet the criteria for the Rastelli operation because of a hypoplastic PA. Consequently, this patient underwent repeat palliative PA augmentation with TEVG as the second palliative surgery and was awaiting a Rastelli operation at the time of this study. This patient was referred to our institution with the unifocalization and palliative RVOT reconstruction already performed in a previous institution. His PA was found to have multiple stenotic regions, especially in the right area. Therefore, we first performed PA augmentation using autologous pericardium and TEVG mold implantation. However, bronchus compression-induced postoperative PA re-stenosis was observed (Figure 3, *A*). Two years after the previous operation, right PA augmentation with TEVG was performed.

**Video 2:**
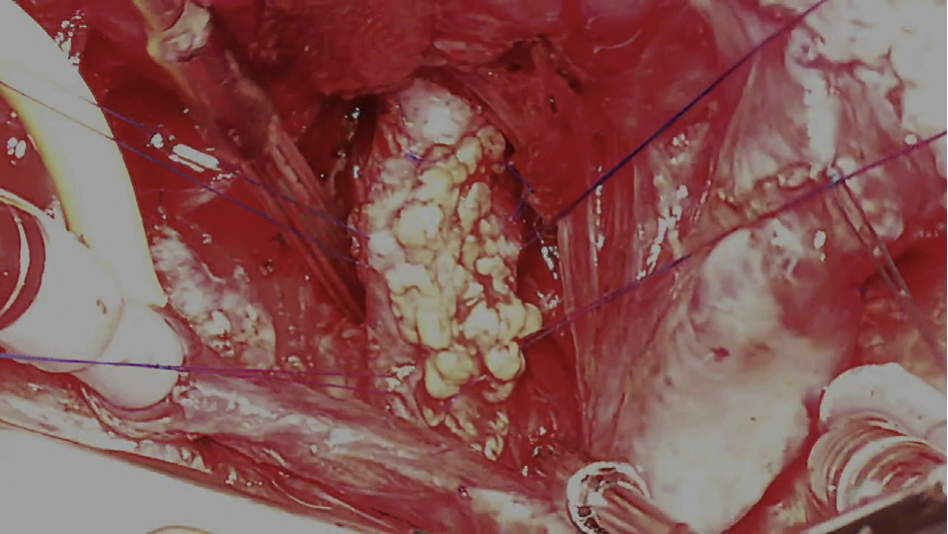
Pulmonary artery augmentation using tissue-engineered vascular graft. Video available at: https://www.jtcvs.org/article/S2666-2507(22)00016-5/fulltext.

**Video 3:**
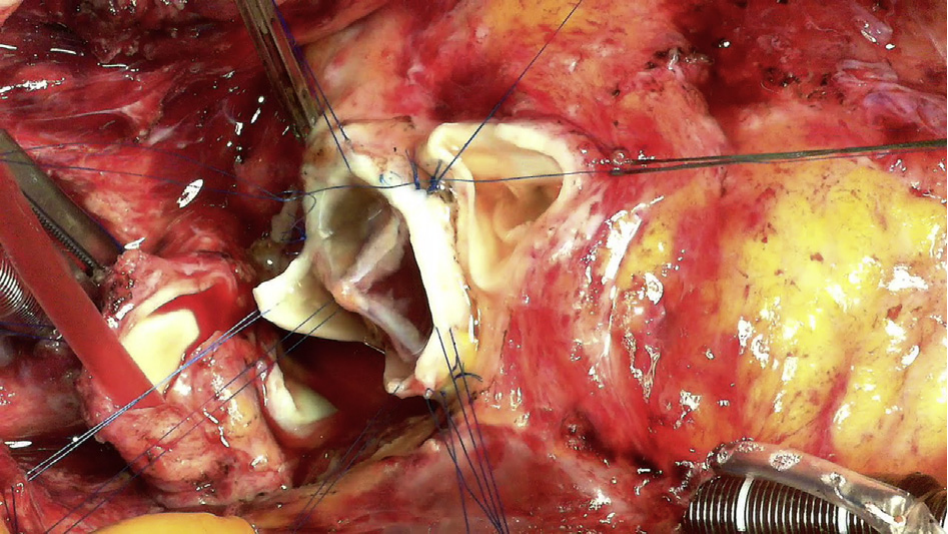
Aortic valvuloplasty using tissue-engineered vascular graft. Video available at: https://www.jtcvs.org/article/S2666-2507(22)00016-5/fulltext.

In group B, both patients were univentricular candidates because of their complex intracardiac morphology. One patient underwent PA augmentation in a Fontan operation, and another underwent palliative RVOT reconstruction and was awaiting the next palliative surgery at the time of this study.

In group C, a half-turned truncal switch operation^5^ was performed when the patient was 3 months old. The postoperative course was good; however, leaflet shortening-induced aortic regurgitation gradually worsened over the course of 8 years. Therefore, AVP was performed with TEVG at 9 years of age, approximately 1 year after TEVG mold implantation.

Anticoagulant therapy generally consisted of 2 mg/kg of acetylsalicylic acid. Moreover, warfarin was prescribed at least 6 months postoperatively, because we did not have any data on the patients' anticoagulant usage status after TEVG implantation.

## Results

**Figure 4:**
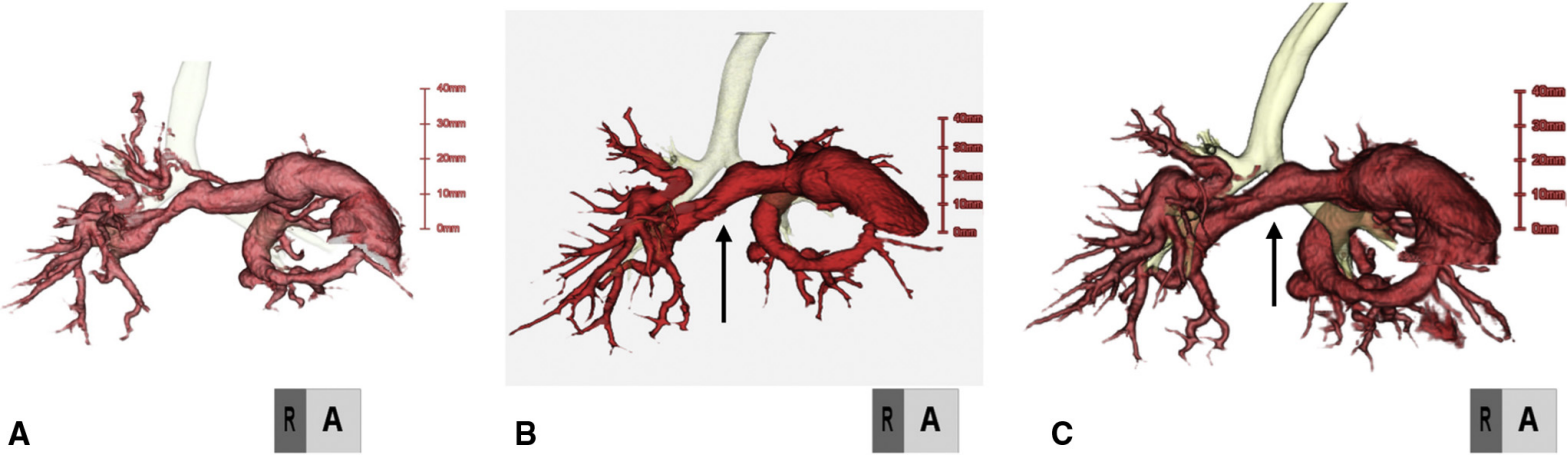
Pre- and postoperative 3-dimensional cardiac computed tomography scan of patient 1. A, Preoperative image. B, Image taken a week after the operation. C, Image taken 40 months postoperatively. Tissue-engineered vascular graft was implanted at the stenotic region of the pulmonary artery (*black arrow*).

**Figure 5:**
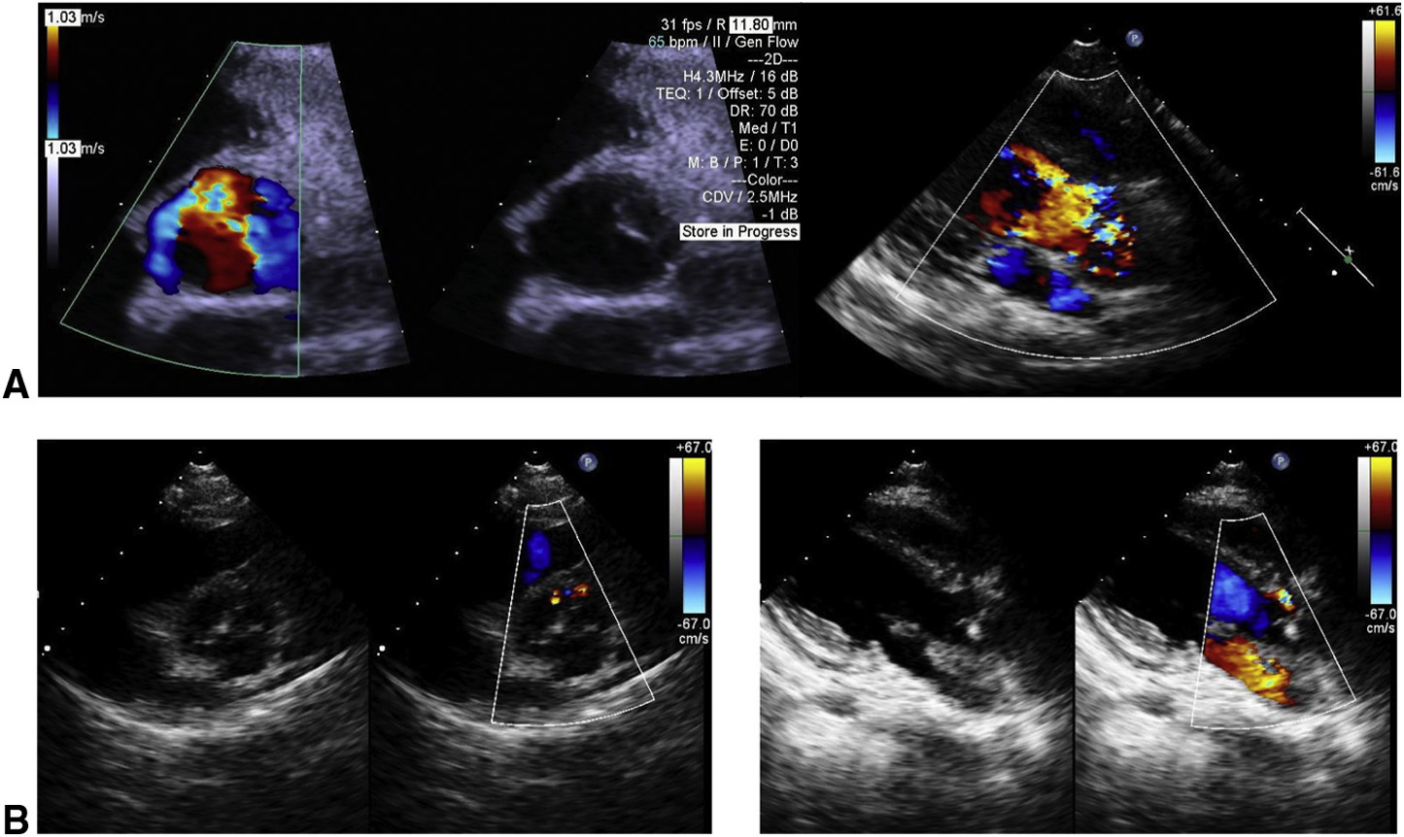
Pre- and postoperative cardiac echography image of patient 7. A, Preoperative image. Moderate to severe aortic regurgitation due to valve leaflet shortening was observed. B, Image taken approximately 3 months postoperatively. Aortic regurgitation improved to trivial to mild regurgitation.

The median follow-up period was 14.4 (range, 3-39.6) months. There were no early or late mortalities. In group A, the postoperative PA configuration was satisfactorily dilated in 3 patients, as shown in 3-dimensional cardiac computed tomography (Figure 4). In the patient who underwent right PA augmentation due to bronchus compression-induced PA re-stenosis (Figure 3, *A*), repeated PA augmentation with TEVG and mold implantation were performed and a good PA configuration was obtained (Figure 3, *B*). Finally, he underwent the Rastelli operation, and PA augmentation with TEVG was performed intraoperatively in a manner similar to that in the previous operation (Figure 3, *C* and *D*). In group B, both patients had a good postoperative PA configuration and no pulmonary hypertension. In all 6 cases with PA augmentation, postoperative hemorrhage from TEVG, aneurysmal change, infection, and thrombosis were not observed. The systolic PA pressures before and after TEVG implantation for each patient in group A are shown in Table 1. In group C, the function of the reconstructed aortic valve was good; only trivial to mild regurgitation and tiny stenoses were found in the neoaortic valve, and decreased leaflet flexibility due to leaflet thickening was not observed (Figure 5).

**Table 1:** Transition of systolic pulmonary arterial pressure in each patient in group A

Patient	Preoperative PAP, mm Hg	Second operation	Postoperative PAP, mm Hg	Third operation	Postoperative PAP, mm Hg	Fourth operation	Postoperative PAP, mm Hg
1	52	PA plasty with TEVG Rastelli	37	–	–	–	–
2	30	PA plasty with TEVG Rastelli	46	–	–	–	–
3	77	PA plasty with TEVG Palliative Rastelli	58	–	–	–	–
4	70	PA plasty with TEVG	65	PA plasty with TEVG Palliative Rastelli	50	PA plasty with TEVG Rastelli	49∗

*PAP*, Pulmonary arterial pressure; *PA*, pulmonary artery; *TEVG*, tissue-engineered vascular graft.

∗ For patient 4, the PAP estimated from cardiac echography was used instead, because catheterization after the Rastelli operation had not yet been performed.

**Figure 6:**
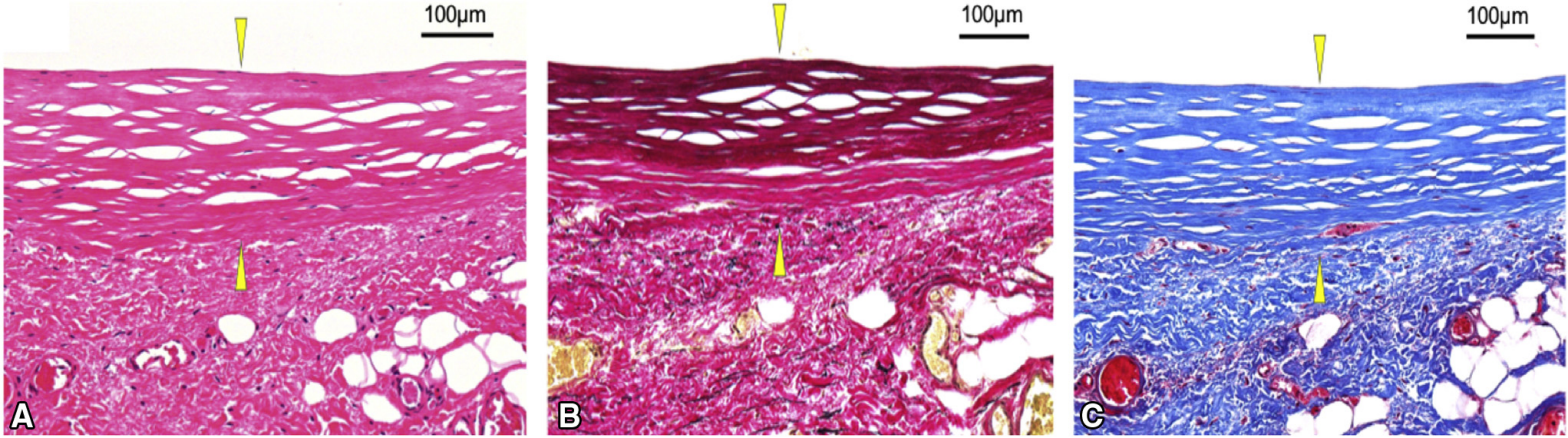
The cross-section was stained with hematoxylin and eosin (A), Masson trichrome (B), and Elastica Van Gieson (C). The *yellow arrow heads* define the depth of the graft walls. The Masson trichrome stain shows that the wall comprises collagen-rich tissue. Elastica Van Gieson staining shows the absence of elastic fiber networks.

On pathological examination, the TEVGs had smooth luminal surfaces. The TEVG wall mainly consisted of collagen fibers and a few fibroblasts; however, elastic fiber formation and/or smooth muscle cells were not observed (Figure 6).

## Discussion

**Figure 7:**
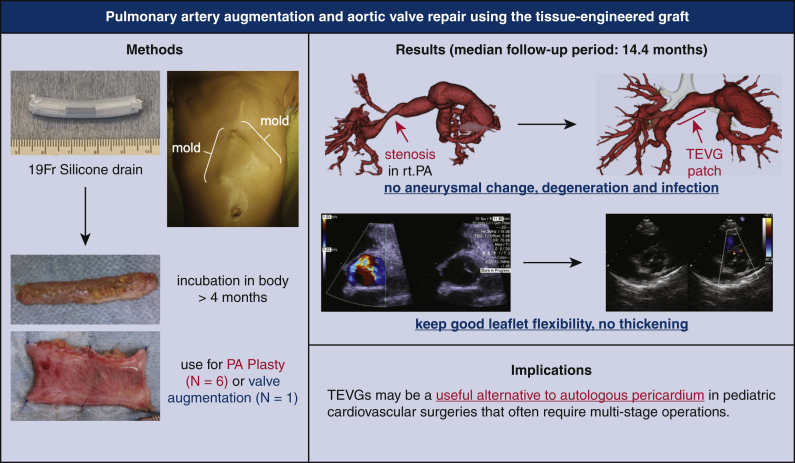
Graphical abstract of this report. *19F*, 19-French; *rt.*, right; *PA*, pulmonary artery; *TEVG*, tissue-engineered vascular graft.

The study is summarized in Figure 7. In cardiovascular surgery, tissue engineering has attracted increasing attention in the past few decades. Some recent studies have indicated that it is possible to provide a TEVG that will remain patent in vivo for substantial use. Such TEVGs have been developed in vitro and are made of materials such as collagen gel,^6^ biodegradable scaffolds within dynamic bioreactors,^7^ nondegradable polyurethane scaffolds,^8^ and so on. However, in pediatric patients, we must consider the growth-associated size mismatch that occurs between TEVGs and the native vessels. Therefore, in this study, we focused on the clinical application of perfectly autologous TEVGs. Our TEVGs have autologous vascular prostheses that are on the basis of the tissue-encapsulation phenomenon of foreign materials in living bodies, which is considered as a biological defense mechanism.^9^^,^^10^

Because the follow-up period in this study was short, we could not reach a consensus on whether TEVGs have a growth potential. However, in our longest-term follow-up experience of 3.3 years, no complications (such as aneurysmal dilatation or re-stenosis due to TEVG degeneration) were noted. Only 1 patient had PA re-stenosis after TEVG implantation; however, this was thought to be due to right bronchus posterior compression and was confirmed to not have been caused by TEVG degeneration itself during the subsequent operation. Moreover, TEVG infection was not observed in any patient, which proved our hypothesis that TEVG is resistant to infection because it is composed of autologous tissue.

Furthermore, regarding the strength of TEVGs, Fujita and colleagues^4^ reported the mechanical strength of TEVGs. As for the suture retention strength, it was concluded that TEVG, the internal mammary artery, and the saphenous vein almost had the same strength.^11^^,^^12^ Additionally, Inoue and colleagues^13^ reported that a short duration of ethanol dehydration and glutaraldehyde treatment for crosslinking might improve surgical handling and burst pressure tolerance and successfully modify the mechanical properties of the aforementioned arteries without interfering with tissue regeneration. As for toxicity, glutaraldehyde is difficult to completely rinse off compared with ethanol,^14^ and clinical reports have mentioned that glutaraldehyde toxicity continues for a longer period in clinical settings.^15^^,^^16^ Considering the occurrence of calcification after glutaraldehyde treatment,^16^ our first choice is currently ethanol treatment.

The wall thickness differs between grafts harvested from humans and from other animals. For instance, the wall thickness of our TEVG was approximately 200 μm, whereas that of grafts harvested from rabbits and rats was 76 μm and 56 μm, respectively. Therefore, our TEVGs were more than twice as thick as the other animal grafts.^2^^,^^3^^,^^17^ In this study, we implanted the silicon mold subcutaneously for 6 months to 2 years; however, no associations were noted between the duration of mold implantation and the TEVG wall thickness. Therefore, we believe that the optimal incubation time in the body is at least 4 months.

As for the number of molds to be implanted, because these molds are harvested intraoperatively for use, they are implanted in the upper abdominal space; therefore, in our experience, a maximum of four 3- to 5-cm molds should be allowed in infants. However, we considered that TEVGs could be implanted not only in the upper abdominal space, but also in any subcutaneous space; therefore, it is possible that more TEVGs can be harvested as long as molds can be embedded. Moreover, as for the diameter of the molds to be embedded, a 19-French silicone tube, which is currently used as a drain tube in clinical settings, is generally used because of the risk of skin ulcer formation secondary to pressure on the dermis from the embedding of an excessively thick mold.

In an in vitro study performed by our team, vascular grafts showed tissue regeneration, including complete endothelialization 2 years after implantation.^2^ In vivo, we did not harvest the implanted TEVG patch at reoperation; therefore, we have no histological data on the implanted TEVG patch in clinical settings. Further investigations are thus required.

### Limitation

This was a single-center, retrospective study. Moreover, this study was not comparative; therefore, the superiority of TEVG over other materials, including autologous pericardium, may not be evaluated.

## Conclusions

The results of PA reconstruction and AVP with TEVG were satisfactory. TEVG-related complications, including aneurysmal change, infection, degeneration resulting in re-stenosis, and hemorrhage, were not observed. Although further follow-up will be needed, our study has revealed that TEVGs are useful alternatives to autologous pericardium in pediatric cardiovascular surgeries that often require multistage operations.

### Webcast

You can watch a Webcast of this AATS meeting presentation by going to: https://aats.blob.core.windows.net/media/21%20AM/AM21_C11/AM21_C11_07_updated.m4v.

**Figure:**
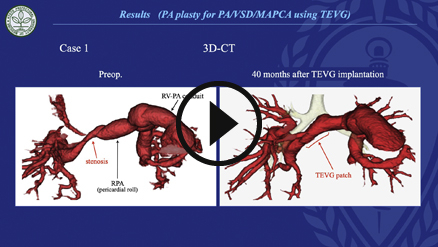

### Conflict of Interest Statement

The authors reported no conflicts of interest.

The *Journal* policy requires editors and reviewers to disclose conflicts of interest and to decline handling or reviewing manuscripts for which they may have a conflict of interest. The editors and reviewers of this article have no conflicts of interest.

## Figures and Tables

**Table E1:** Demographic characteristics patients in group A

	Patient number (group A)			
	1	2	3	4
Diagnosis	PA/VSD/MAPCA			
First operation (our institution)	UF/pRVOTR, **TEVG mold implantation**			PA plasty, **TEVG mold implantation**
Age/BW at first operation	2.0 Years/10.0 kg	1.3 Years/7.3 kg	1.3 Years/7.3 kg	4.8 Years/15.4 kg
Interval	8.9 Months	14.0 Months	6.5 Months	2.2 Years
Second operation	Rastelli *PA plasty with TEVG*		re-pRVOTR *PA plasty with TEVG*	*PA plasty with TEVG*
Age/BW at second operation	2.7 Years/11.6 kg	2.4 Years/9.1 kg	1.8 Years/7.9 kg	7.0 Years/19.0 kg
Interval	–	–	–	7.0 Months
Third operation				re-pRVOTR *PA plasty with TEVG* **TEVG mold implantation**
Age/BW at third operation				7.8 Years/19.6 kg
Interval				1.1 Year
Fourth operation				Rastelli *PA plasty with TEVG*
Age/BW at fourth operation				8.8 Years/21.8 kg
Follow-up from last operation	3.3 Years	1.9 Years	1.2 Years	3.0 Months

TEVG mold implantation was described in bold style, and PA plasty with TEVG was described in italic style.

*PA*, Pulmonary atresia; *VSD*, ventricular septal defect; *MAPCA*, major aortopulmonary collateral arteries; *UF*, unifocalization; *pRVOTR*, palliative right ventricular outflow tract reconstruction; *TEVG*, tissue-engineered vascular graft; *BW*, body weight.

**Table E2:** Demographic characteristics of patients in groups B and C

	Patient number (groups B and C)			
	5	6	7	
Diagnosis	DORV/CoA		TGA/VSD/PS	
First operation	Bilateral PA banding		HTTSO	
Age/BW at first operation	9 Days/3.0 kg	8 Days/2.6 kg	3.0 Months/5.4 kg	
Interval	8.9 Months	2 Months	7.9 Years	
Second operation	Arch repair, DKS pRVOTR	Arch repair MPA banding	**TEVG mold implantation**	
Age/BW at Second operation	2.7 Months/4.6 kg	2.0 Months/3.8 kg	8.0 Years/22.3 kg	
Interval	2.9 Months	1.0 Years	1.1 Years	
Third operation	PA plasty re-pRVOTR	BCPS, DKS PA plasty	*AVP with TEVG*	
Age/BW at third operation	5.1 Months/4.7 kg	1.2 Years/8.6 kg	9.2 Years/24.4 kg	
Interval	1.7 Years	9.8 Months	–	
Fourth operation	**TEVG mold implantation**		–	
Interval	6.0 Months	7.6 Months		
Fifth operation	re-pRVOTR *PA plasty with TEVG*	EC-TCPC *PA plasty with TEVG*		
Follow-up from last operation	6.0 Months	3.0 Months	6.0 Months	

TEVG mold implantation was described in bold style, and PA plasty with TEVG was described in italic style.

*DORV*, Double-outlet right ventricle; *CoA*, coarctation of aorta; *TGA*, transposition of the great arteries; *VSD*, ventricular septal defect; *PS*, pulmonary stenosis; *PA*, pulmonary artery; *HTTSO*, half-turned truncal switch operation; *BW*, body weight; *DKS*, Damus–Kaye–Stansel anastomosis; *pRVOTR*; palliative right ventricular outflow tract reconstruction; *MPA*, main pulmonary artery; *TEVG*, tissue-engineered vascular graft; *BCPS*, bidirectional cavopulmonary shunt; *AVP*, aortic valve plasty; *EC-TCPC*, extracardiac total cavopulmonary connection.

## Supplementary Data

Video 1 Harvest of tissue-engineered vascular graft mold. Video available at: https://www.jtcvs.org/article/S2666-2507(22)00016-5/fulltext.

Video 2 Pulmonary artery augmentation using tissue-engineered vascular graft. Video available at: https://www.jtcvs.org/article/S2666-2507(22)00016-5/fulltext.

Video 3 Aortic valvuloplasty using tissue-engineered vascular graft. Video available at: https://www.jtcvs.org/article/S2666-2507(22)00016-5/fulltext.
